# Supplementary material for: Mutant p53 drives an immune cold tumor immune microenvironment in oral squamous cell carcinoma
Source: Commun Biol. 2022 Jul 28;5:757. doi: 10.1038/s42003-022-03675-4 (PMC9334280; doi:10.1038/s42003-022-03675-4)
Supplement: Supplementary file 2 — Supplemental Information [file 42003_2022_3675_MOESM2_ESM.pdf]

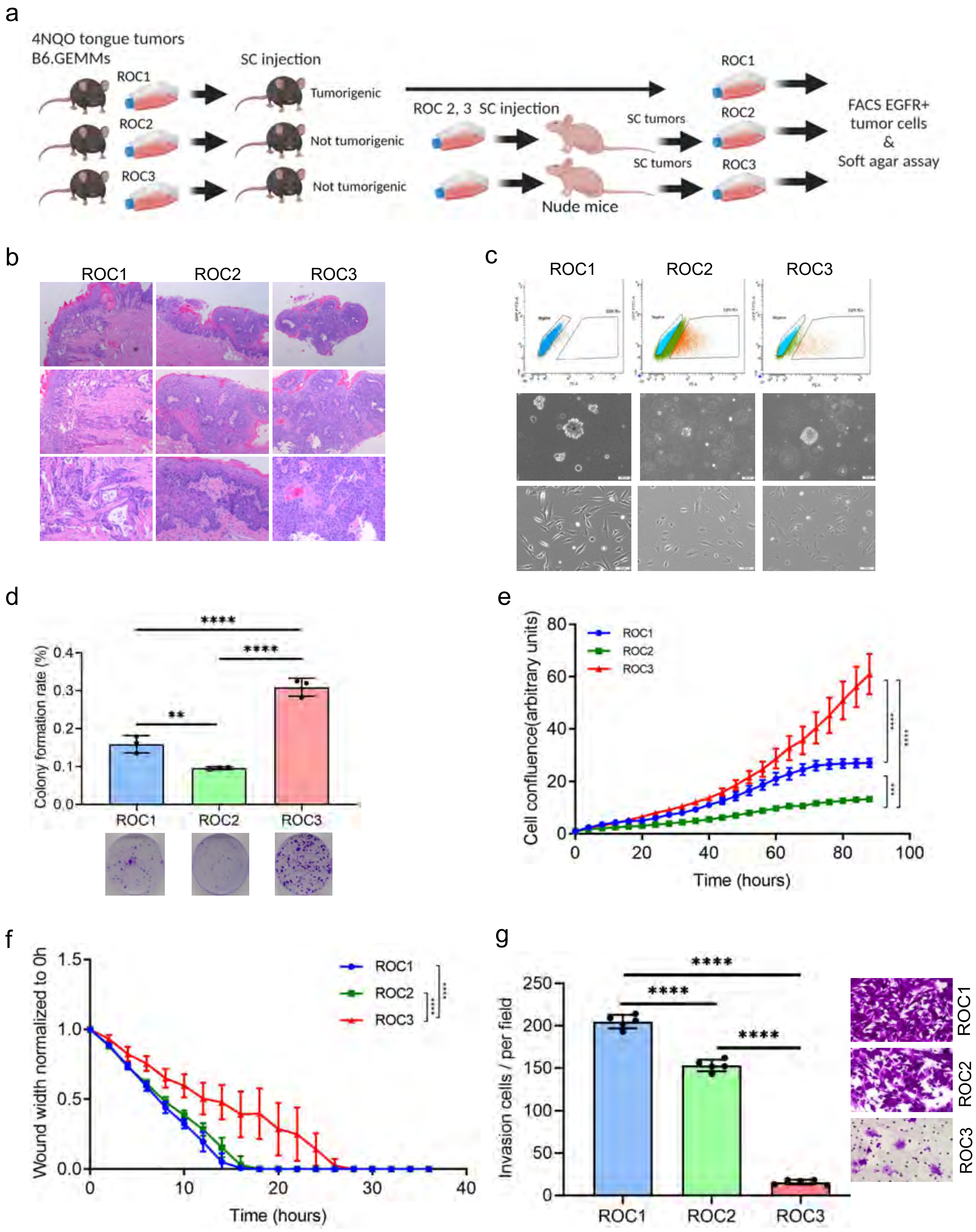

Supplementary Fig. 1. ROC cell line characterization. (**a**) ROC cell line generation strategy diagram. C57BL/6 (B6) backcrossed genetically engineered mouse models (GEMMs) were exposed to 4NQO-carcinogen in the drinking water. Tongue tumors were dissociated and cultured in DMEM, ROC1 showed subcutaneous tumorigenicity in C57BL/6J mice. ROC2 and ROC3 were subcutaneously implanted into athymic nude mice. Tumors were processed and placed in tissue culture; later, fluorescence-activated cell sorting (FACS) of enriched high expression of epidermal growth factor receptor (EGFR) of tumor cells were recovered for functional and tumorigenicity studies. (**b**) Histopathological images of the murine oral carcinomas from which the ROC cell lines were derived. (**c**) FACS of epithelial cells with high EGFR expression (top panel); representative brightfield images of cell colonies in soft agar (middle panel); and representative brightfield images of the ROC cell lines (bottom panel). (**d**) ROC cell lines display differences in colony formation ability. (**e**) Results from cell proliferation assay. (**f**) Results from wound scratch assay. (**g**) Results from cellular invasion assay. $^{**}P < 0.01$, $^{***}P < 0.001$, $^{****}P < 0.0001$, by one-way ANOVA with Tukey's post hoc test.

a

Cell line	Number of cells injected orthotopically			
	10,000	50,000	100,000	500,000
ROC 1	60%	40%	40%	80%
ROC 2	ND	ND	ND	ND
ROC 3	ND	ND	ND	60%

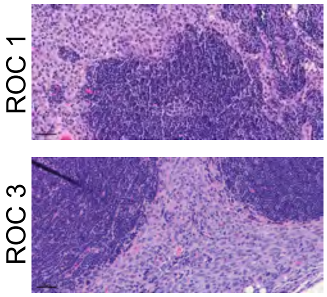

b

Cell line	Number of cells injected orthotopically			
	10,000	50,000	100,000	500,000
ROC 1	ND	ND	ND	ND
ROC 2	ND	ND	ND	ND
ROC 3	ND	ND	ND	ND

c

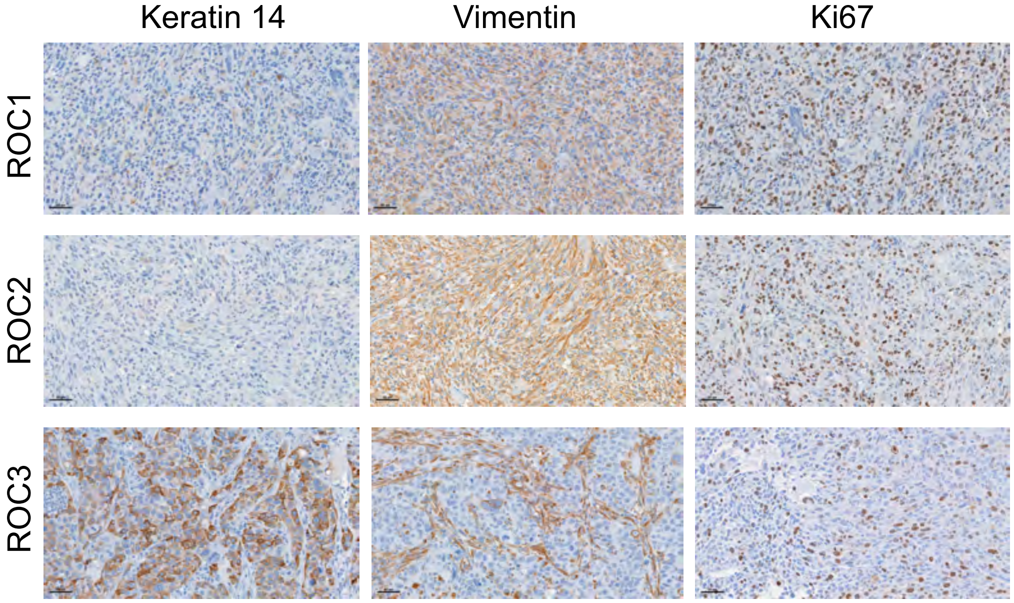

Supplementary Fig. 2. ROC cell lines show different lymph node metastasis frequencies and different expression of EMT markers. (**a**) Orthotopic ROC tumors in C57BL/6 mice show different incidences of cervical lymph node metastasis. (**b**) Orthotopic ROC tumors in C57BL/6 mice show no incidences of lung metastasis. ND Not detected. (**c**) Staining for K14, vimentin, and Ki67 expression. Scale bars, 50 μm.

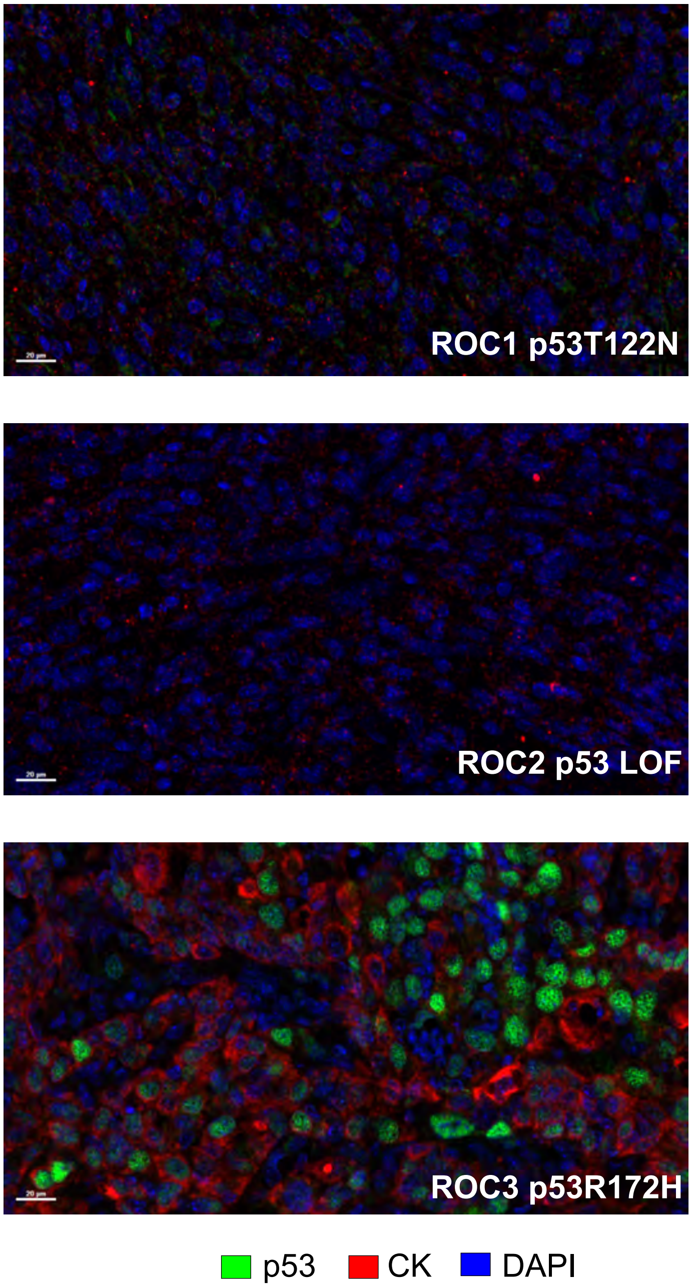

Supplementary Fig. 3. Opal multiplex IHC studies of p53 in ROC cell lines. ROC1-3 tumor tissues were subjected to multiplex IHC to detect p53 (green), cytokeratin (red), and DAPI (blue). Scale bars, 20 μm.

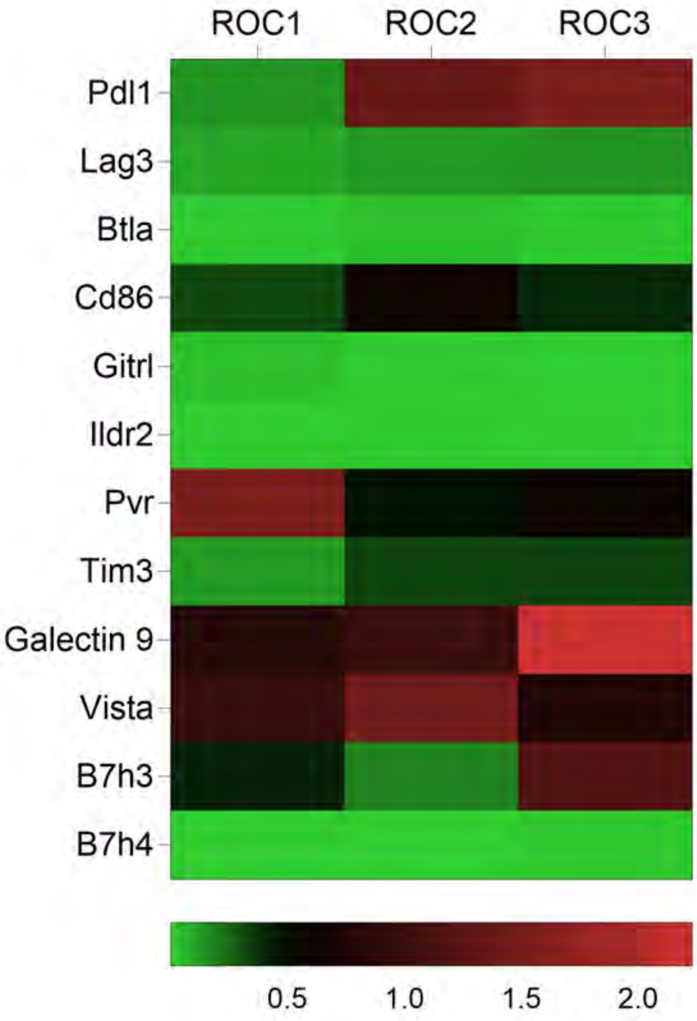

Supplementary Fig. 4. Gene expression analysis of immune checkpoint inhibitors in the ROC cell lines. Heatmaps represent high (red) and low (green) RNA expression by arbitrary units (color bar ranges from 0 to 2.22).

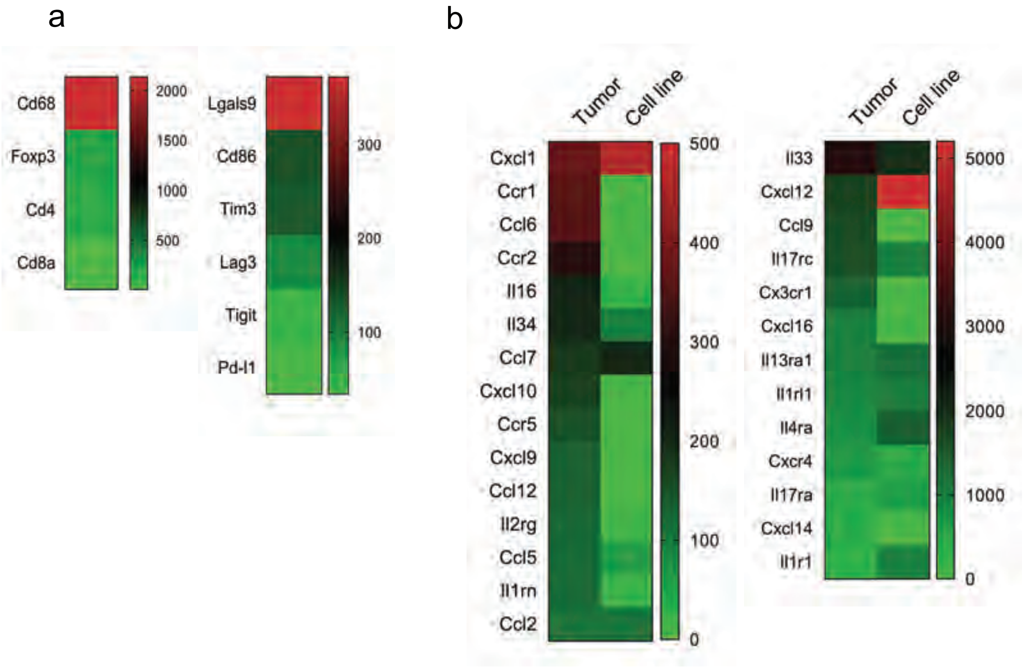

Supplementary Fig. 5. Gene expression analysis of immune cell markers, cytokines, and chemokines in the ROC1 tumors and cell line. (**a**) Normalized RNA expression of immune cell markers and immune checkpoints in transcripts per million (TPM). (**b**) Normalized RNA expression of cytokines and chemokines in TPM. Heatmaps represent high (red) and low (green) RNA expression by arbitrary units; in (**a**), color bars range from 0 to 2136 and from 34 to 371; in (**b**), color bars range from 0 to 500 and from 0 to 5200.

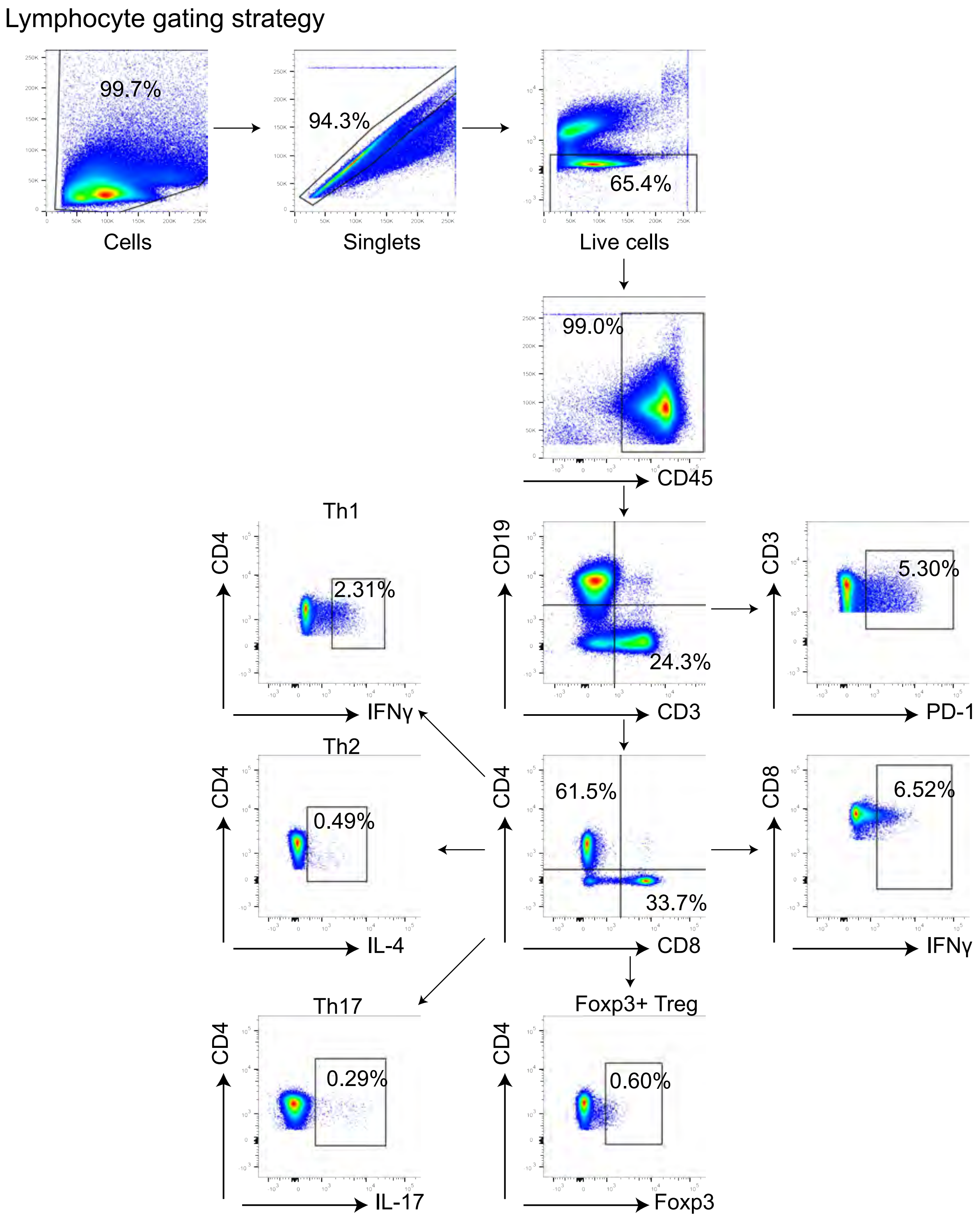

Supplementary Fig. 6 The flow cytometry gating strategy for lymphocytes.

Myeloid cells gating strategy

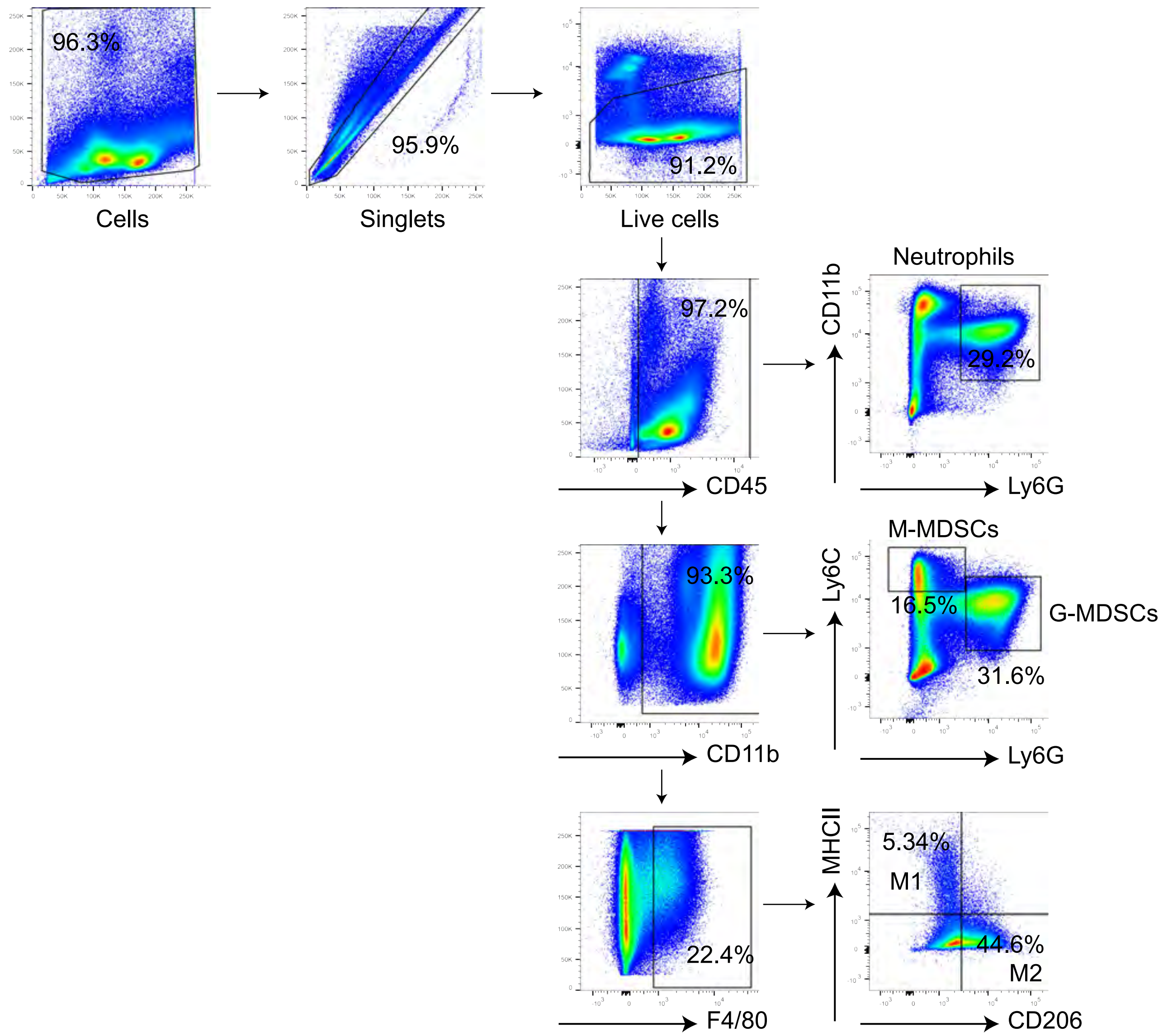

Supplementary Fig. 7 The flow cytometry gating strategy for myeloid cells.

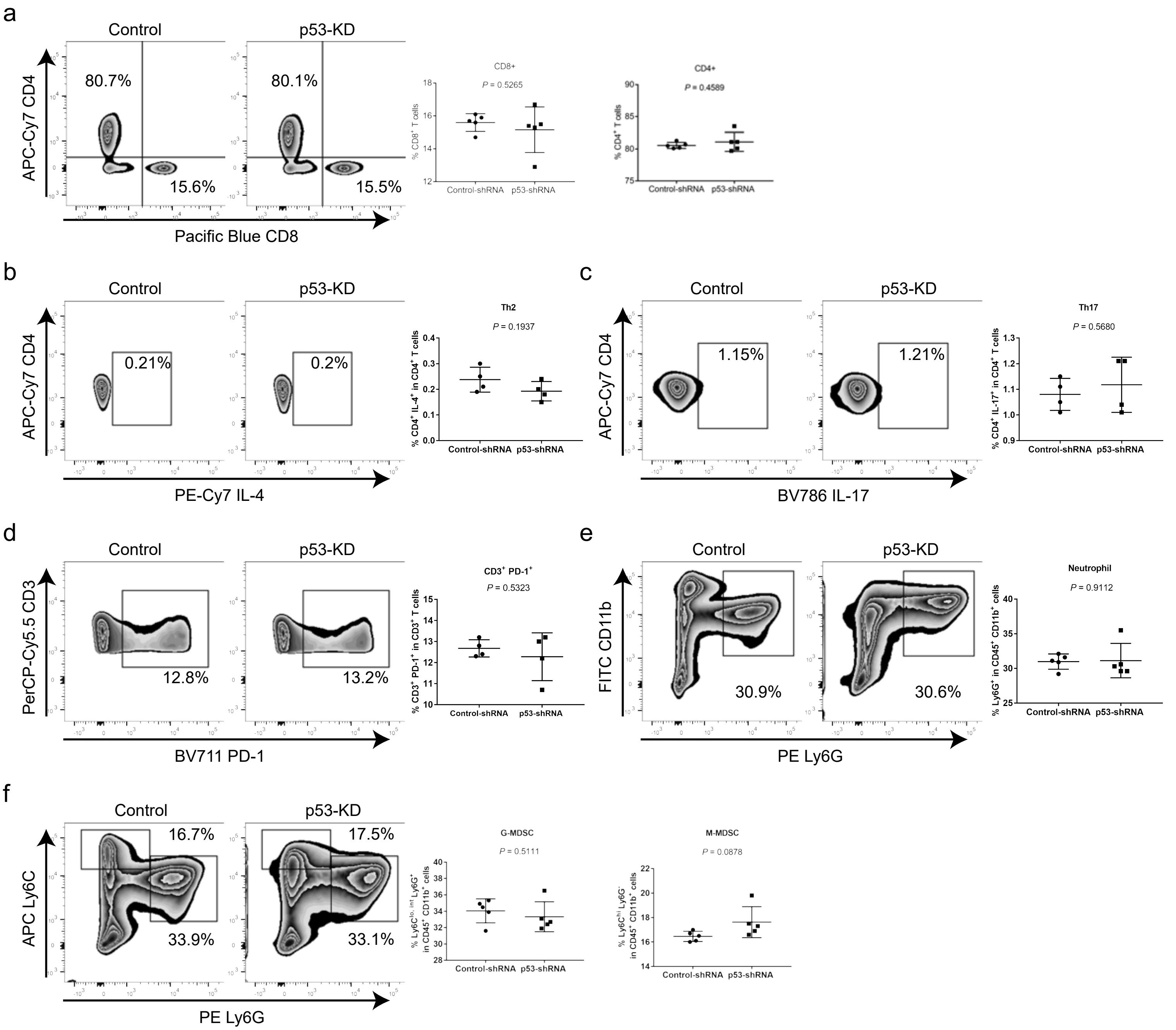

Supplementary Fig. 8. Flow cytometry of immune cell populations not altered by ROC1-conditioned media. (**a**) Comparison of the frequencies of CD8+ and CD4+ cells among T cells in splenocytes. (**b**) Comparison of the frequencies of IL4+ cells among CD4+ T cells (Th4 cells) in splenocytes. (**c**) Comparison of the frequencies of IL-17+ cells among CD4+ T cells (Th17 cells) in splenocytes. (**d**) Comparison of the frequencies of PD-1+ cells among CD3+ T cells in splenocytes. (**e**) Comparison of the frequencies of Ly6G+ CD11b- cells (neutrophils) in bone marrow cells (BMCs). (**f**) Comparison of the frequencies of LyG+ LyC^{lo},^{int} cells (granulocyte-myeloid derived suppressor cells [G-MDSCs]) and LyG- LyChigh cells (monocytic- myeloid derived suppressor cells [M-MDSCs]) in BMCs.

Supplementary Materials and Methods

Cell sorting

Tumor cell monolayers were mobilized with Accutase cell detachment medium (Thermo Fisher, #00-4555-56) and resuspended in a density of 1–2 × 10^7 cells/mL. Cells (1 × 10^7) were stained with a phycoerythrin (PE)-conjugated anti-EGFR antibody (GeneTex, #GTX20231) or a PE-conjugated anti–IgG2a κ isotype control antibody (BioLegend, #400508) and analyzed with a BD FACSAria II flow cytometer (BD Biosciences) using FACSDiva version 6.1.3 software. Living cells were identified by forward scatter/side scatter gating.

Soft agar colony formation assay

For the colony formation assay, in each well of a 6-well plate, 1 mL of 0.6% soft agarose in medium containing 10% FBS served as a base layer, on top of which was placed 0.3% soft agar, 20% FBS, mixed with suspended cells (1 × 10^5 cells/well). The cells were maintained in an incubator at 37°C in 5% CO_2 for 14-28 d until formed colonies were visible. The cells were supplemented with 100 μL of complete medium every 3 d. The visible colonies were counted, and images of the colonies were captured with use of a Leica microscope. Each experiment was performed on three replicate samples and repeated three times. The largest of each of the three cell lines' colonies was selected for cell culture to obtain the final three ROC1-3 cell lines. DNA of these colonies were sent for p53 Sanger sequencing.

Clone formation assay

For the colony formation assay, cells were seeded in 6-cm plates at a density of 1500 cells/plate. The cell culture was performed for 2 wk and was terminated when macroscopic colonies were visible. Cells were harvested, washed with PBS, and then fixed with 10% neutral-buffered formalin for 30 min. The cells were then stained with 0.1% crystal violet for 30 min. After they were washed and air-dried, the clones were manually counted. Each assay was performed in triplicate.

Cell proliferation assay

Cells were seeded in 96-well plates at a density of 2×10^3 cells/well in 8 replications. Photomicrographs of the cells were taken every 4 h by using an IncuCyte live cell imager (Essen Biosciences, Ann Arbor, MI), and the confluence of the cultures was measured for more than 96 h with use of IncuCyte software (Essen Biosciences).

Wound scratch assay

Cells were seeded in 96-well plates at a density of 2×10^4 cells/well in 8 replications. Wounds were generated by using WoundMaker (Essen Biosciences) according to the manufacturer's instructions when cells reached 100% confluence. Growth medium was replaced with serum-free medium, and the wound confluences were monitored and photographed every 2 h with the IncuCyte real-time live-cell analysis system.

Matrigel invasion assay

Cell invasion was assessed by using 6.5-mm transwell chambers (Corning Coster, Corning, NY) coated with Matrigel (BD Biosciences, San Jose, CA) according to the manufacturer's protocol. Cells were plated at a density of 1×10^5 cells/chamber in 3 replications.

In vivo orthotopic mouse model and treatment

Cells were mixed with Matrigel (BD Biosciences, #354230) at a 1:1 ratio. To determine whether ROC1-3 cell lines would grow into tumors when implanted at the primary site of origin, we injected 1×10^4, 5×10^4, 1×10^5, and 5×10^5 cells in 30 µL of buffered solution into the tongues of C57BL/6 mice (5 mice per group).

To assess mutant p53 T122N's regulation of different tumor-associated immune populations, we injected 5×10^4 ROC1 cells (parental, control-shRNA, p53-shRNA) into C57BL/6 and beige mice.

Mice were treated with anti–PD-1 and/or anti-TIGIT antibodies beginning 5 days after inoculation with 50,000 ROC1 cells. Mice were randomized to receive intraperitoneal injections of the IgG2a isotype control (BioCell, #BE0083), 200 µg of anti–PD-1 antibody (BioCell, #BE0146), 200 µg of anti-TIGIT antibody (BioCell, #BE0274), or combination therapy three times per week for 3 wk.

Mice were treated with the STING agonist c-di-GMP (Invivo Gen, #tlrl-nacdg) once the diameter of their tumors increased to 2-3 mm. Mice were randomized to receive intratumoral injections of 30 µL of PBS or 25 µL of c-di-GMP in 30 µL of PBS once every 3 d;

injections were given three times.

Mice received an anti–PD-1 antibody plus c-di-GMP once the diameter of their tumors increased to 2-3 mm. Mice were randomized and grouped into control and therapy groups. Anti–PD-1 and c-di-GMP were injected once every 3 d and given 6 times, except for the fourth and sixth c-di-GMP doses, which were skipped.

Tumor sizes were measured twice a week after treatment. Tumor volume was calculated by using the equation tumor length × tumor width^2/2. Tumor-bearing mice were humanely killed by CO_2 asphyxiation. Tongue tumors, cervical lymph nodes, and lungs were surgically excised and photographed. Tissues were embedded in paraffin and sliced into 5-μm-thick sections. Pathological examinations of the cervical lymph nodes and lungs were performed to assess metastasis.

Immunohistochemical analysis

The primary antibodies used for immunohistochemical analysis (IHC) were antibodies against CD8a (Cell Signaling, 1:100, #98941), CD4 (Cell Signaling, 1:100, #25229), FoxP3 (eBioscience, 1:100, #14-5773-82), CD68 (Abcam, 1:200, #ab125212), CD11c (Cell Signaling, 1:100, #97585), CD206 (Abcam, 1:2000, #ab64693), PD-1 (Cell Signaling, 1:100, #84651), CTLA-4 (Biorbyt Ltd, 1:500, #orb253158), TIGIT (Millipore Sigma, 1:1500, #ZRB1454), vimentin (Cell Signaling, 1:200, #5741), Ki67 (Abcam, 1:1000, #ab15580), and cytokeratin 14 (ThermoFisher Scientific, 1:800, #PA5-167222).

Fluorescent multiplex IHC consecutive staining on a single slide

The primary antibodies used for multiplex IHC were antibodies against p53 (Leica, 1:200, #P53-PROTEIN-CM5), pan-cytokeratin (Abcam, 1:100, #ab27988), CD8a (Cell Signaling, 1:100, Cat# 98941), CD4 (Cell Signaling, 1:100, #25229), FoxP3 (eBioscience, 1:100, #14-5773-82), granzyme B (Cell Signaling, 1:100, #46890), CD68 (Abcam, 1:150, #ab125212), CD206 (Abcam, 1:2000, #ab64693), PD-1 (Cell Signaling, 1:100, #84651), and STING (Cell Signaling, 1:100, #13647).

Western blotting

The primary antibodies used for Western blotting were antibodies against p53 (Cell Signaling, 1:1000, #32532) and β-actin (Santa Cruz Biotechnology, 1:5000, #sc81178).

Supplementary Tables

Supplementary Table 1. Sequencing read mapping summary

Cell line	Read length	Total read pair	% Mapped
ROC1	100	124482837	99.61
ROC2	100	122458097	99.62
ROC3	100	118393342	99.58

Supplementary Table 2. Single-nucleotide variant counts

	No. (%)		
**Genomic alteration location**	**ROC1**	**ROC2**	**ROC3**
Downstream	4 (0.10)	5 (0.13)	11 (0.10)
Exonic	1445 (37.86)	1402 (36.10)	4704 (44.84)
Intergenic	473 (12.39)	479 (12.33)	821 (7.83)
Intronic	1505 (39.43)	1579 (40.65)	3970 (37.85)
ncRNA_exonic	142 (3.72)	151 (3.89)	232 (2.21)
ncRNA_intronic	90 (2.36)	105 (2.70)	198 (1.89)
Splicing	8 (0.21)	7 (0.18)	104 (0.99)
Upstream	17 (0.45)	23 (0.59)	47 (0.45)
Upstream;downstream	1 (0.03)	1 (0.03)	4 (0.04)
UTR3	83 (2.17)	89 (2.29)	268 (2.55)
UTR5	49 (1.28)	43 (1.11)	131 (1.25)
Total	3817 (100)	3884 (100)	10490 (100)

Supplementary Table 3. INDEL counts

Genomic alteration location	No. (%)		
	ROC1	ROC2	ROC3
Downstream	3 (0.28)	2 (0.17)	1 (0.09)
Exonic	35 (3.22)	34 (2.96)	59 (5.29)
Intergenic	68 (6.26)	75 (6.52)	88 (7.89)
Intronic	870 (80.04)	927 (80.61)	850 (76.23)
ncRNA_exonic	11 (1.01)	15 (1.3)	18 (1.61)
ncRNA_intronic	40 (3.68)	36 (3.13)	31 (2.78)
Splicing	10 (0.92)	9 (0.78)	11 (0.99)
Upstream	5 (0.46)	5 (0.43)	3 (0.27)
UTR3	33 (3.04)	37 (3.22)	33 (2.96)
UTR5	12 (1.10)	10 (0.87)	21 (1.88)
Total	1087 (100)	1150 (100)	1115 (100)

Supplementary Table 4. Variable allele frequency and depth of the mutated genes in the ROC cell lines

Chromosome	NCBI_Build	Start_Position	End_Position	Reference_Allele	Tumor_Seq_Allele2	Hugo_Symbol	Variant_Classification	tx	exon	txChange	aaChange	Variant_Type	Cell line_Sample_Barcode	Func.refGene	Gene.refGene	GeneDetail.refGene	ExonicFunc.refGene	AAChange.refGene	DP_ReadDepth_Phred15	RD_Depth_Reference	AD_Depth_Variant	FREQ_VAF	VAF	PVAL
chr11	GRCm38	69588512	69588512	G	A	Trp53	Missense_Mutation	NM_001127233	exon5	c.G515A	p.R172H	SNP	ROC3	exonic	Trp53	.	nonsynonymous SNV	Trp53:NM_001127233:exon5:c.G515A:p.R172H,Trp53:NM_011640:exon5:c.G515A:p.R172	29	0	29	1	1	3.33E-17
chr8	GRCm38	44952269	44952269	G	T	Fat1	Missense_Mutation	NM_001081286	exon1	c.G2056T	p.E686X	SNP	ROC3	exonic	Fat1	.	stopgain	Fat1:NM_001081286:exon1:c.G2056T:p.E686X	122	67	55	0.4508	0.45	9.81E-21
chr8	GRCm38	45009949	45009949	T	C	Fat1	Missense_Mutation	NM_001081286	exon4	c.T3802C	p.S1268P	SNP	ROC3	exonic	Fat1	.	nonsynonymous SNV	Fat1:NM_001081286:exon4:c.T3802C:p.S1268P	100	75	25	0.25	0.25	5.36E-09
chr2	GRCm38	26468266	26468266	C	G	Notch1	Missense_Mutation	NM_008714	exon25	c.G4552C	p.G1518R	SNP	ROC3	exonic	Notch1	.	nonsynonymous SNV	Notch1:NM_008714:exon25:c.G4552C:p.G1518R	46	26	20	0.4348	0.43	6.72E-08
chr2	GRCm38	26481639	26481639	C	T	Notch1	Missense_Mutation	NM_008714	exon5	c.G797A	p.C266Y	SNP	ROC3	exonic	Notch1	.	nonsynonymous SNV	Notch1:NM_008714:exon5:c.G797A:p.C266Y	32	11	21	0.6562	0.66	3.14E-09
chr15	GRCm38	48011074	48011074	G	T	Csmd3	Missense_Mutation	NM_001081391	exon14	c.C2125A	p.Q709K	SNP	ROC3	exonic	Csmd3	.	nonsynonymous SNV	Csmd3:NM_001081391:exon14:c.C2125A:p.Q709K	62	2	60	0.9677	0.97	1.33E-33
chr3	GRCm38	59077141	59077141	G	C	Med12l	Missense_Mutation	NM_177855	exon10	c.G1466C	p.W489S	SNP	ROC3	exonic	Med12l	.	nonsynonymous SNV	Med12l:NM_177855:exon10:c.G1466C:p.W489S	131	100	31	0.2366	0.24	6.21E-11
chr15	GRCm38	98849975	98849975	C	A	Kmt2d	Missense_Mutation	NM_001033276	exon35	c.G9467T	p.R3156L	SNP	ROC3	exonic	Kmt2d	.	nonsynonymous SNV	Kmt2d:NM_001033276:exon35:c.G9467T:p.R3156L	92	0	92	1	1	6.94E-55
chr15	GRCm38	98855063	98855063	C	G	Kmt2d	Missense_Mutation	NM_001033276	exon26	c.G5477C	p.G1826A	SNP	ROC3	exonic	Kmt2d	.	nonsynonymous SNV	Kmt2d:NM_001033276:exon26:c.G5477C:p.G1826A	161	7	154	0.9565	0.96	1.74E-84
chr5	GRCm38	4064639	4064639	G	C	Akap9	Missense_Mutation	NM_194462	exon39	c.G9917C	p.G3306A	SNP	ROC2	exonic	Akap9	.	nonsynonymous SNV	Akap9:NM_194462:exon39:c.G9917C:p.G3306A	86	44	42	0.4884	0.49	2.76E-16
chr9	GRCm38	15919234	15919234	G	T	Fat3	Missense_Mutation	NM_001080814	exon24	c.C12963A	p.N4321K	SNP	ROC3	exonic	Fat3	.	nonsynonymous SNV	Fat3:NM_001080814:exon24:c.C12963A:p.N4321K	82	18	64	0.7805	0.78	2.11E-29
chr9	GRCm38	16375309	16375309	C	G	Fat3	Missense_Mutation	NM_001080814	exon1	c.G2917C	p.D973H	SNP	ROC3	exonic	Fat3	.	nonsynonymous SNV	Fat3:NM_001080814:exon1:c.G2917C:p.D973H	110	40	70	0.6364	0.64	4.87E-29
chr3	GRCm38	38949642	38949642	C	A	Fat4	Missense_Mutation	NM_183221	exon3	c.C5509A	p.H1837N	SNP	ROC3	exonic	Fat4	.	nonsynonymous SNV	Fat4:NM_183221:exon3:c.C5509A:p.H1837N	162	103	59	0.3642	0.36	2.61E-21
chr14	GRCm38	54948454	54948454	G	T	Myh6	Missense_Mutation	NM_001164171	exon31	c.C4363A	p.L1455M	SNP	ROC3	exonic	Myh6	.	nonsynonymous SNV	Myh6:NM_001164171:exon31:c.C4363A:p.L1455M,Myh6:NM_010856:exon31:c.C4363A:p	42	31	11	0.2619	0.26	0.00023

Supplementary Table 5. STR profile analysis of murine oral cancer cell lines

Marker #	ROC1	ROC2	ROC3	MOC-1	MOC-2
MCA-1-1	15	15,16	15,16	16	16
MCA-1-2	19	19	19	19	18,19
MCA-2-1	16	16	16	16	16
MCA-3-2	14	14	14	14	14
MCA-4-2	20.3	20.3	20.3	20.3	20.3
MCA-5-5	17	17, 18	17	18	17
MCA-6-4	18	18	18, 19	18	18
MCA-6-7	17	15, 17	15	15	15
MCA-7-1	27.2	26.2, 27.2	26.2, 27.2	25.2	26.2
MCA-8-1	16	16	16	16	16
MCA-9-1	18	18	17, 18	18	18, 19
MCA-11-2	16	16	16	16	16
MCA-12-1	17	17	17	17	17
MCA-13-1	17	17	17	17	17
MCA-15-3	22.3	22.3	22.3	22.3	22.3, 23.3
MCA-17-2	15, 16	16	16	15, 16	15
MCA-18-3	16	16	16	16	17
MCA-19-2	13	13	13, 14	13	13
MCA-X-1	26, 27	26	26, 27	27	26

MOC-1 and MOC-2 were generated as reported [54].

Supplementary Table 6. RNAseq read mapping summary

Sample	Triplicate Sample	Read length	Total read pair	Map %
ROC1 cell line	ROC1A	150	21334612	89.10%
	ROC1B	150	24377416	88.90%
	ROC1C	150	25054994	85.50%
ROC1 tumor	T7203	150	24120945	89.70%
	T7210	150	22917360	86.70%
	T7211	150	25162567	88.90%

Supplementary Table 7. qPCR Oligonucleotides information

Gene	Forward (5' - 3')	Reverse (5' - 3')
Ccl2	AGGTGTCCCAAAGAAGCTGT	GACCTTAGGGCAGATGCAGTT
Ccl5	AGCTGCCCTCACCATCATCCTCACT	CACTTCTTCTCTGGGTTGGCACAC
Cxcl5	CGGAGCTGCGTTGTGTTTG	TTCCGCTTAGCTTTCTTTTTGTCA
Cxcl16	TGAGGCTGAGGCAAATGAGAAACA	GAAGACAATGGCCAGGAGGGACAG
Il6	CACTTCACAAGTCGGAGGCT	GCCACTCCTTCTGTGACTCC
Il17rc	AGAGGAGTTGCTGCAAGACT	CCCATAGCGATCCCATGTTG
Il33	TCGGGTACCAAGCATGAAGAGAAC	TCCACACCGTCGCCTGATTG
Il1rl1	ATAAGGCACACCATAAGGCTGAGA	ATCGTTCCGGGTTTTGTAAGGTGT
GADPH	GGAGAGTGTTTCCTCGTCCC	ACTGTGCCGTTGAATTTGCC

Supplementary Table 8. Antibodies used in flow cytometry

Antibody	Clone	Fluorescence
**Lymphocyte panel**		
Ghost Dye™	--	AmCyan
CD45	104	AF700
CD3	17A2	PerCP-Cy5.5
CD4	GK1.5	APC-Cy7
CD8	53-6.7	PB
CD19	1D3	APC
IFN-γ	XMG1.2	FITC
IL-4	BVD6-2462	PE-Cy7
IL-17A	TC11-18H10	BV786
FoxP3	FJK-16S	PE
PD-1	RMP1-30	BV711
**Myeloid panel**		
Ghost Dye™	--	AmCyan
CD45	104	AF700
CD11b	M1/70	FITC
CD11c	N418	PE-Cy7
Ly6C	HK1.4	APC
Ly6G	1A8	PE
CD206	C068C2	BV421
MHC II	M5/114.15.2	BV711

Additional Supplementary Information

Uncropped images corresponding to Figure 1e

ROC1

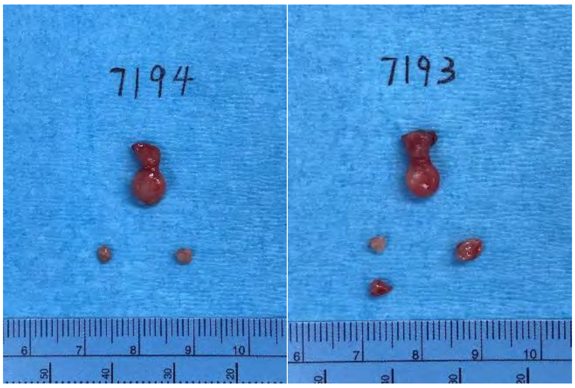

ROC2

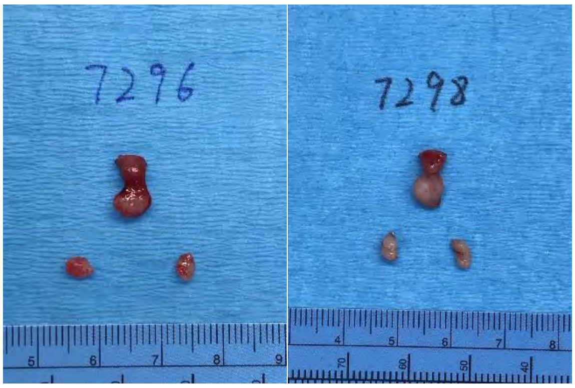

ROC3

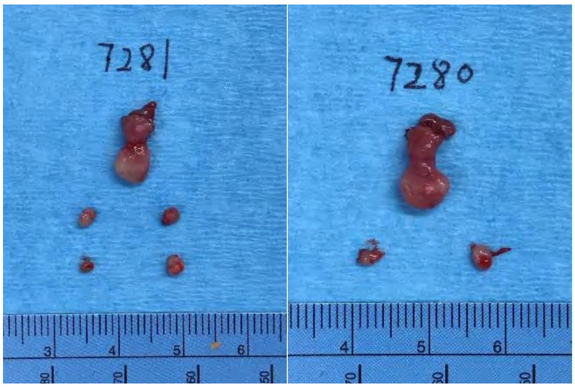

Uncropped blot corresponding to Figure 4a

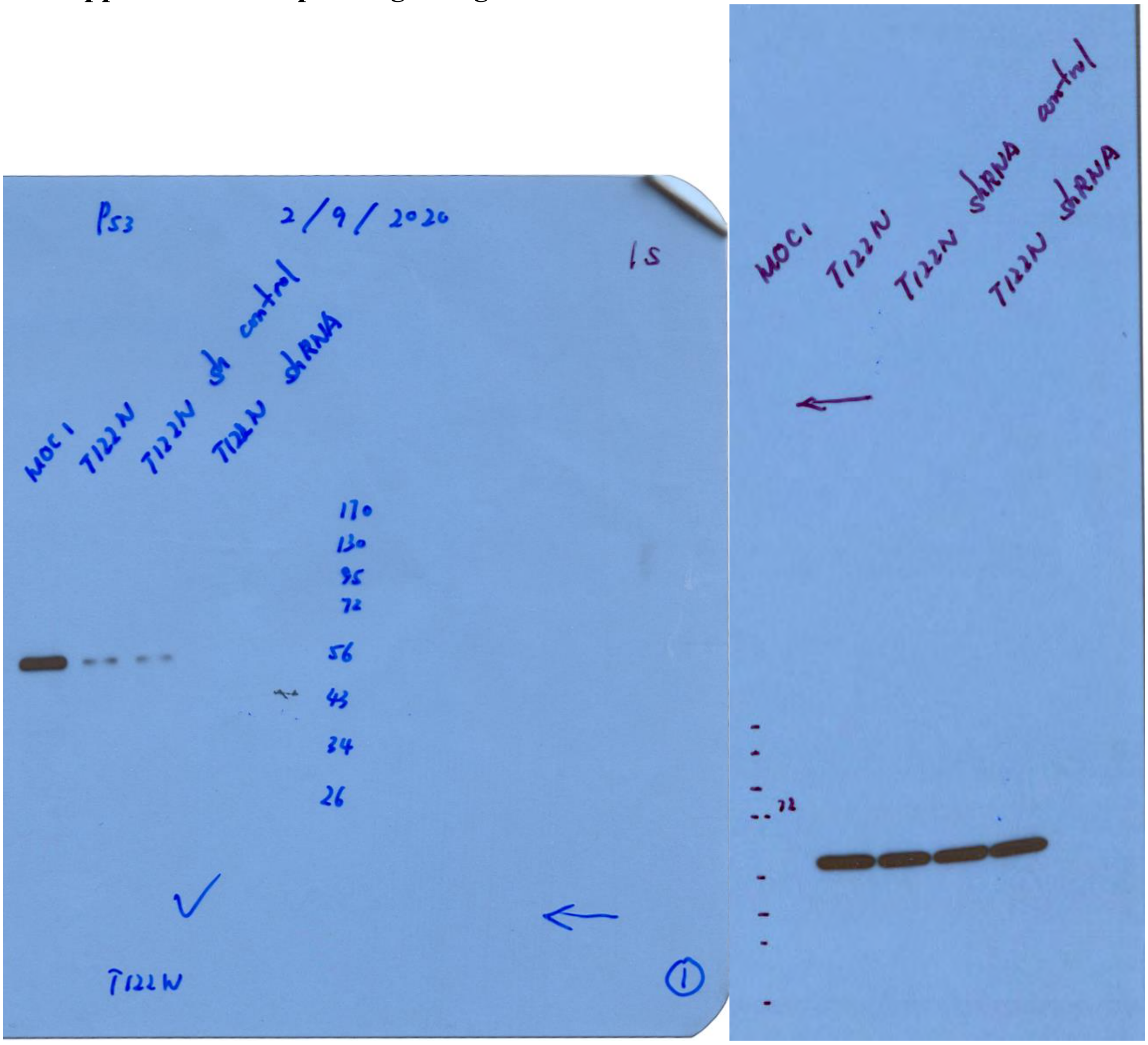

Uncropped image corresponding to Figure 5

a

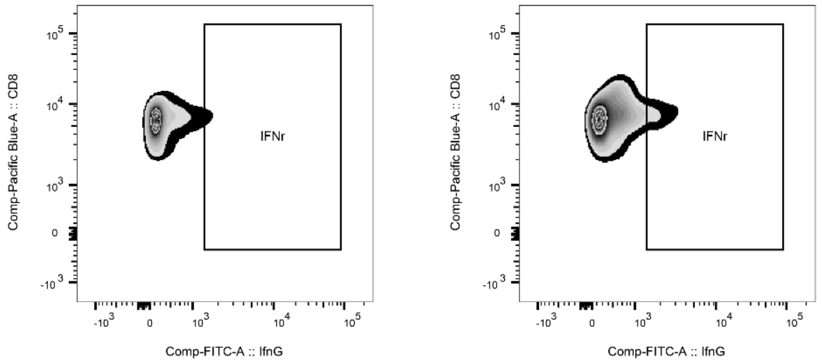

b

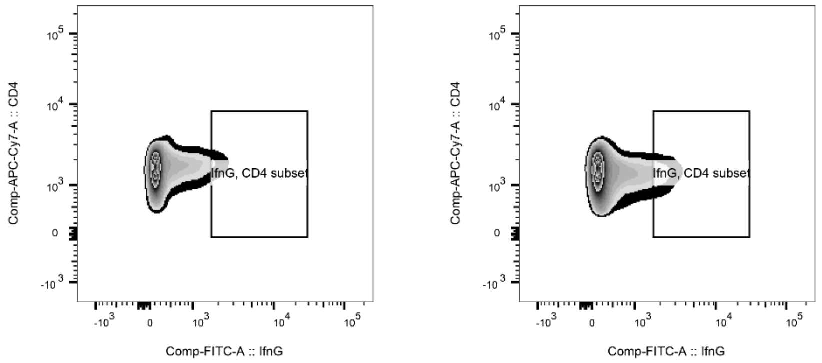

c

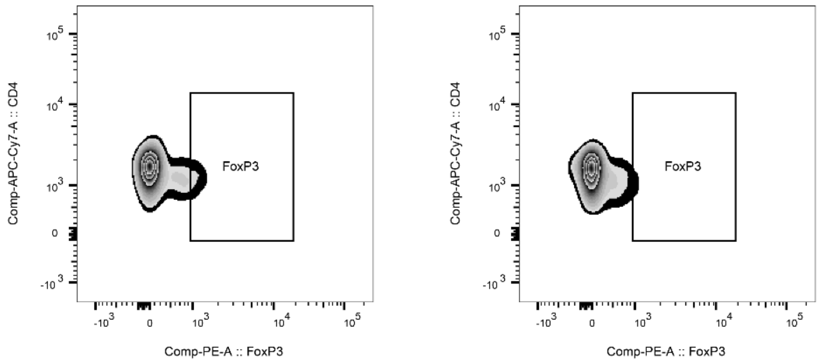

d

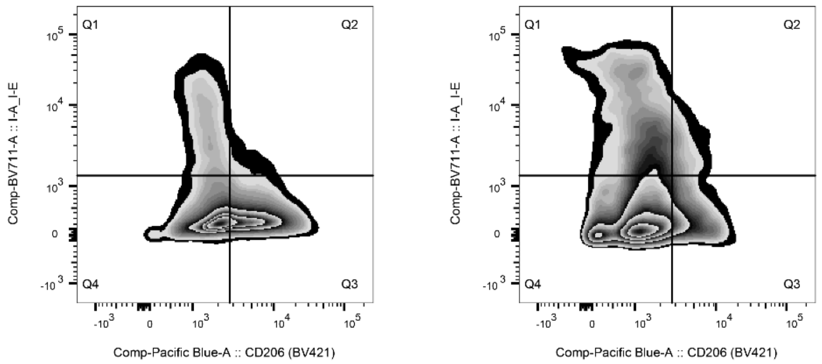

Uncropped image corresponding to Supplementary Fig. 6

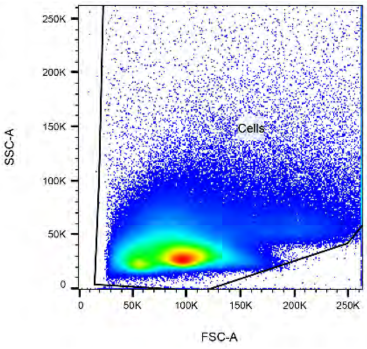

Cells

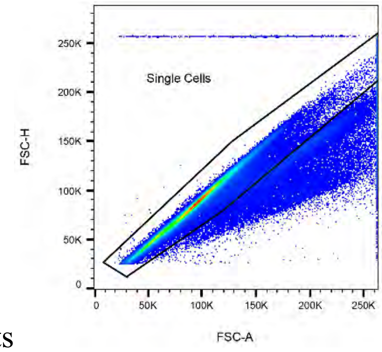

Singlets

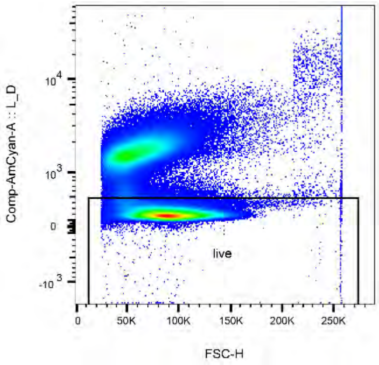

Live cells

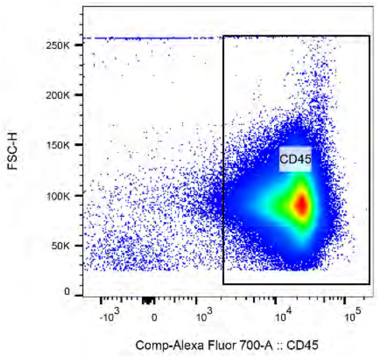

CD45

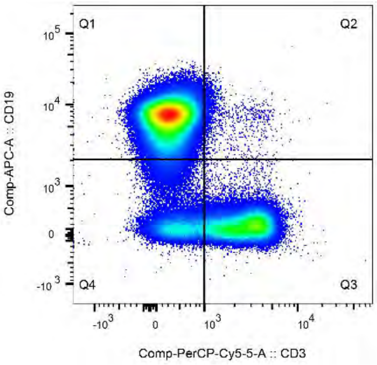

CD3-CD19

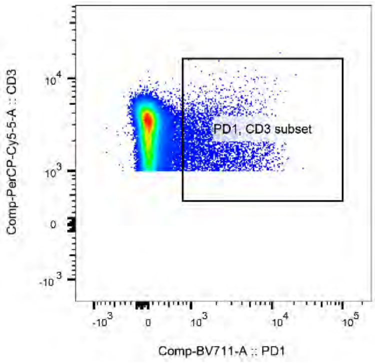

CD3-PD-1

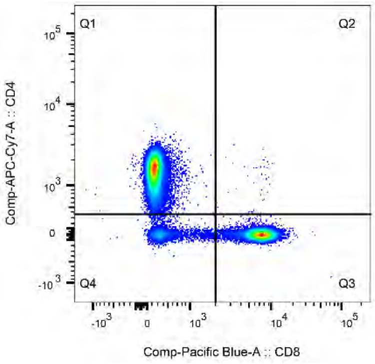

CD4-CD8

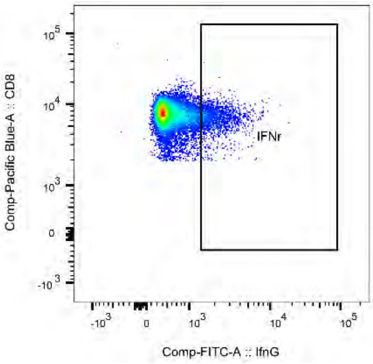

CD8-IFNγ

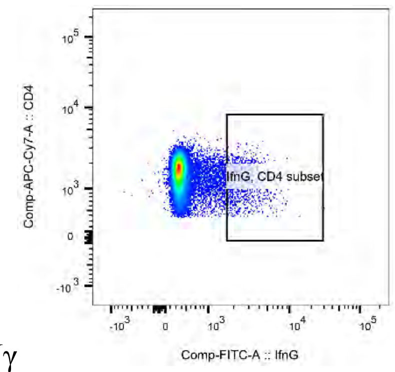

CD4-IFNγ

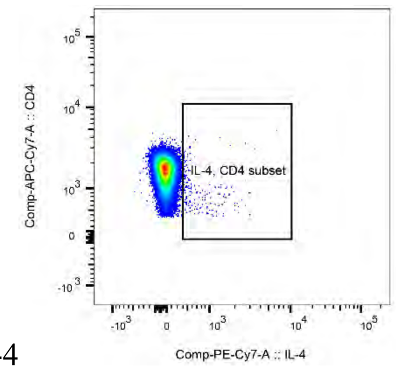

CD4-IL-4

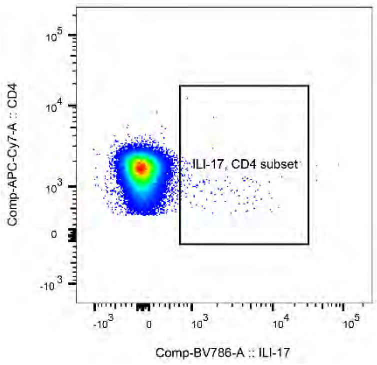

CD4-IL-17

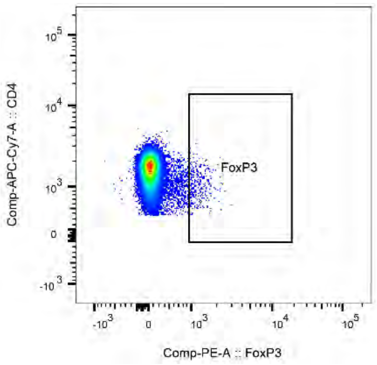

CD4-Foxp3

Uncropped image corresponding to Supplementary Fig. 7

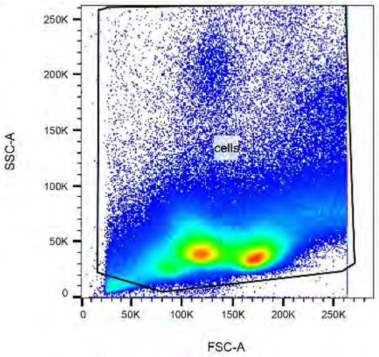

Cells

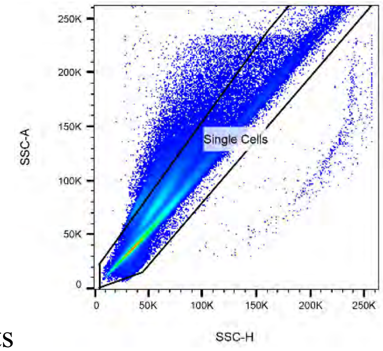

Singlets

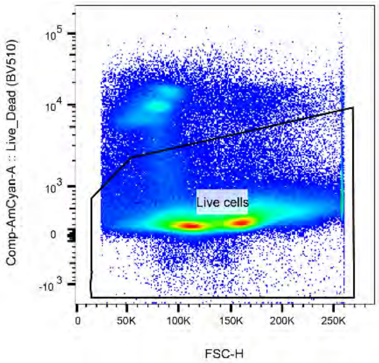

Live cells

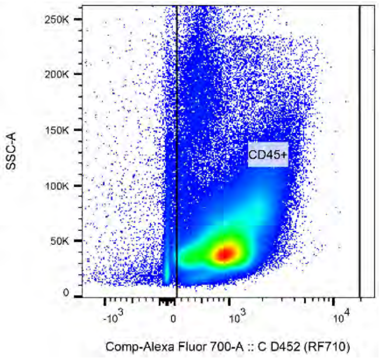

CD45

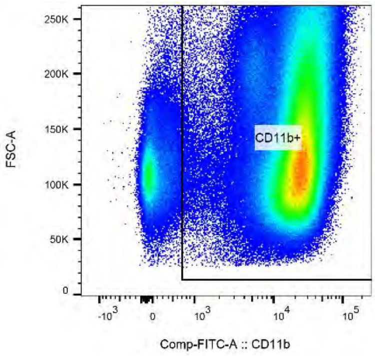

CD11b

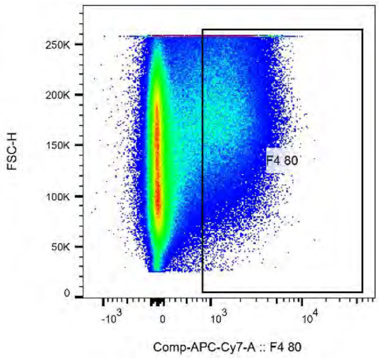

F4/80

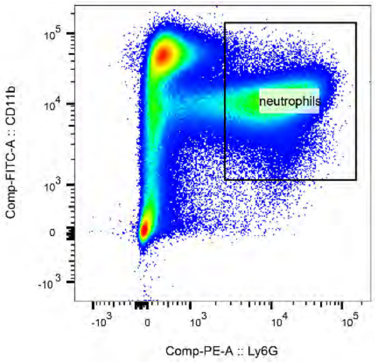

Neutrophils

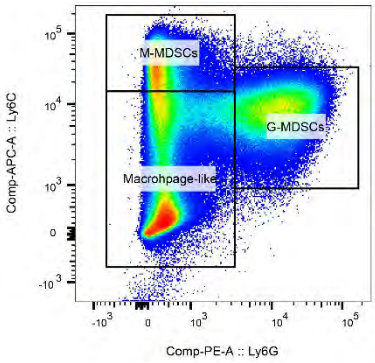

MDSC

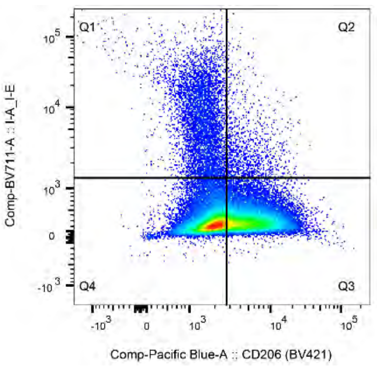

M1-M2

Uncropped image corresponding to Supplementary Fig. 8

a

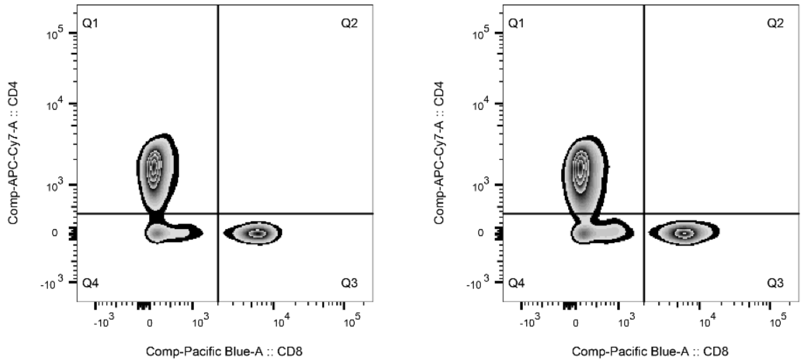

b

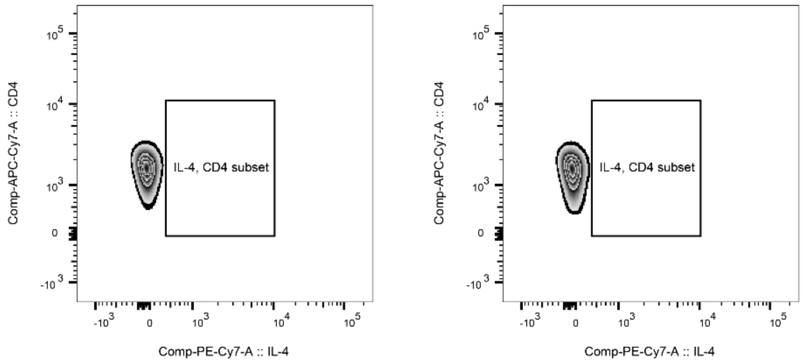

c

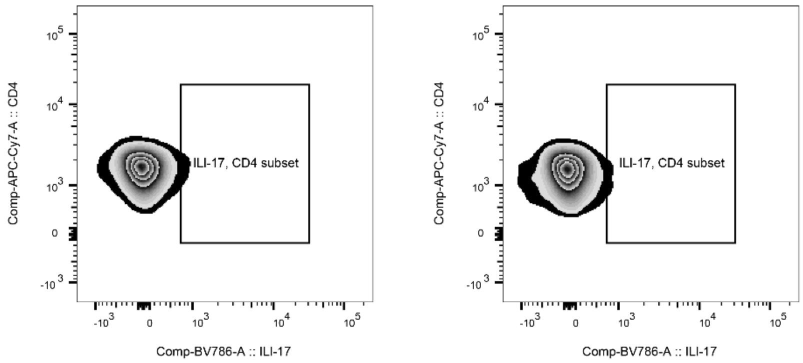

d

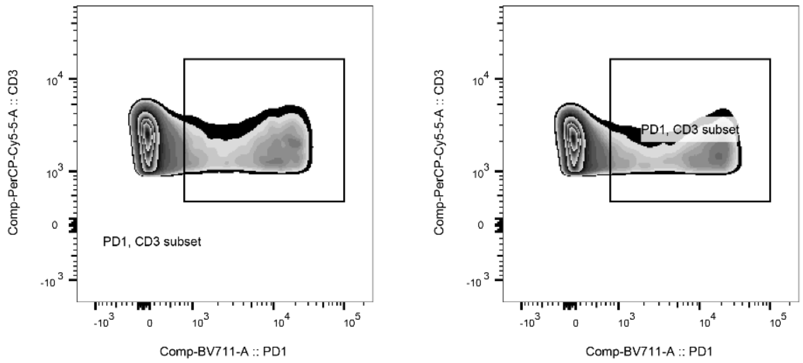

e

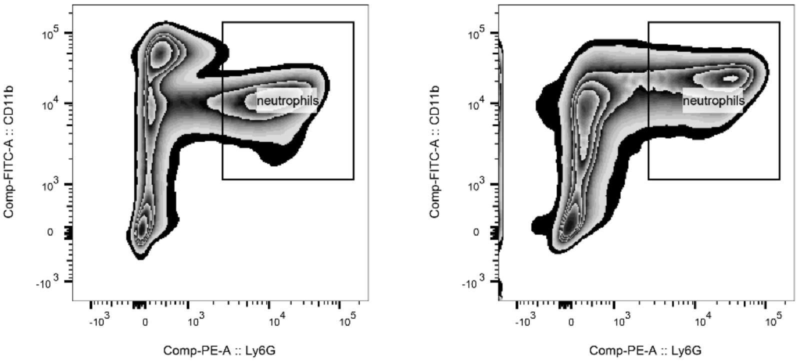
neutrophils
neutrophils
Comp-FITC-A :: CD11b
Comp-FITC-A :: CD11b
Comp-PE-A :: Ly6G
Comp-PE-A :: Ly6G

f

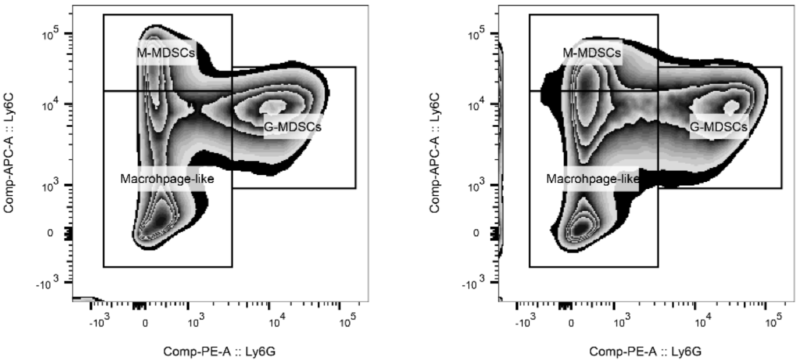
M-MDSCs
G-MDSCs
Macrohpage-like
M-MDSCs
G-MDSCs
Macrohpage-like
Comp-APC-A :: Ly6C
Comp-APC-A :: Ly6C
Comp-PE-A :: Ly6G
Comp-PE-A :: Ly6G
