# Supplementary material for: Mutant p53 drives an immune cold tumor immune microenvironment in oral squamous cell carcinoma
Source: Commun Biol. 2022 Jul 28;5:757. doi: 10.1038/s42003-022-03675-4 (PMC9334280; doi:10.1038/s42003-022-03675-4)
Supplement: Supplementary file 3 — Description of Additional Supplementary Files [file 42003_2022_3675_MOESM3_ESM.pdf]

Description of Additional Supplementary Files

File name: Supplementary Data 1
Description: Pathway analysis of ROC1 p53-KD cells.
