# Supplementary material for: Mutant p53 drives an immune cold tumor immune microenvironment in oral squamous cell carcinoma
Source: Commun Biol. 2022 Jul 28;5:757. doi: 10.1038/s42003-022-03675-4 (PMC9334280; doi:10.1038/s42003-022-03675-4)
Supplement: Supplementary file 5 — Reporting Summary [file 42003_2022_3675_MOESM5_ESM.pdf]

nature portfolio

Corresponding author(s): Roberto Rangel

Last updated by author(s): Mar 18, 2022

Reporting Summary

Nature Portfolio wishes to improve the reproducibility of the work that we publish. This form provides structure for consistency and transparency in reporting. For further information on Nature Portfolio policies, see our Editorial Policies and the Editorial Policy Checklist.

Statistics

For all statistical analyses, confirm that the following items are present in the figure legend, table legend, main text, or Methods section.

n/a	Confirmed	
☐	☒	The exact sample size (n) for each experimental group/condition, given as a discrete number and unit of measurement
☐	☒	A statement on whether measurements were taken from distinct samples or whether the same sample was measured repeatedly
☐	☒	The statistical test(s) used AND whether they are one- or two-sided<br>*Only common tests should be described solely by name; describe more complex techniques in the Methods section.*
☒	☐	A description of all covariates tested
☐	☒	A description of any assumptions or corrections, such as tests of normality and adjustment for multiple comparisons
☐	☒	A full description of the statistical parameters including central tendency (e.g. means) or other basic estimates (e.g. regression coefficient) AND variation (e.g. standard deviation) or associated estimates of uncertainty (e.g. confidence intervals)
☐	☒	For null hypothesis testing, the test statistic (e.g. F, t, r) with confidence intervals, effect sizes, degrees of freedom and P value noted<br>*Give P values as exact values whenever suitable.*
☒	☐	For Bayesian analysis, information on the choice of priors and Markov chain Monte Carlo settings
☒	☐	For hierarchical and complex designs, identification of the appropriate level for tests and full reporting of outcomes
☒	☐	Estimates of effect sizes (e.g. Cohen's d, Pearson's r), indicating how they were calculated

Our web collection on statistics for biologists contains articles on many of the points above.

Software and code

Policy information about availability of computer code

Data collection	We used the reference genome of GRCm38 of Mus musculus strain C57BL/6J by using TopHat2 (v2.1.1) to analyze our RNAseq and whole exome sequencing data.

Data analysis

- Whole exome sequencing and RNA-seq data were generated by the BGI Genomics Company. The RNA-seq reads were mapped against the reference genome of GRCm38 of Mus musculus strain C57BL/6J by using TopHat2 (v2.1.1), and the mapped reads per gene were counted by using HTSeq (v0.11.0) based on gene annotation of GENCODE M19 (Supplementary Table 5). The reads counts were scaled/normalized by transcripts per million (TPM). WES reads were mapped by using bwa (with default parameter setting) to against the mouse genome assembly mm10. Nucleotide mutations were called by using Varscan2 (parameter setting: --strand-filter 1 --min-coverage 30 --p-value 0.01 --min-freq-for-hom 0.9 --output-vcf 1 --variants 1) based on the output generated by samptools mpileup of bam files. The VCF files of mutations for down-streaming analysis were annotated by the refGene database and filtered by the snp142 database.
- Fluorescent multiplex IHC consecutive staining on a single slide were analyzed using multispectral imaging for quantitative unmixing of many fluorophores and tissue autofluorescence.
- Reverse transcription-quantitative PCR (RT-qPCR). We used Bio-Rad CFX96 Touch Real-Time PCR Detection System and the data was analyzed using Bio-Rad CFX manager 3.1 (Version 3.1.1517.08323), and revised using of the following formula: RQ = 2-ΔΔCq. Each reaction was performed in triplicate.
- Statistical analysis was performed using GraphPad Prism (Version 9.0.0) and SPSS 19.0 (Chicago, IL)
- Fluorescence-activated cell sorting (FACS) All data were acquired with using an LSRFortessa X-20 flow cytometer (BD Biosciences) and analyzed with FlowJo software (Tree Star).
- Immunohistochemical quantification was defined as the density of cells and average optical density per view (20X; n = 3 stained tumors; every sample had 5 views).

For manuscripts utilizing custom algorithms or software that are central to the research but not yet described in published literature, software must be made available to editors and reviewers. We strongly encourage code deposition in a community repository (e.g. GitHub). See the Nature Portfolio guidelines for submitting code & software for further information.

Data

Policy information about availability of data

All manuscripts must include a data availability statement. This statement should provide the following information, where applicable:

- Accession codes, unique identifiers, or web links for publicly available datasets
- A description of any restrictions on data availability
- For clinical datasets or third party data, please ensure that the statement adheres to our policy

All data generated or analyzed during this study are included in this publication article as supplementary information files. Raw data can be obtained from authors upon reasonable request

Field-specific reporting

Please select the one below that is the best fit for your research. If you are not sure, read the appropriate sections before making your selection.

☒ Life sciences ☐ Behavioural & social sciences ☐ Ecological, evolutionary & environmental sciences

For a reference copy of the document with all sections, see nature.com/documents/nr-reporting-summary-flat.pdf

Life sciences study design

All studies must disclose on these points even when the disclosure is negative.

Sample size	For all experiments n=3 was selected as the minimal replicate number. For experiments involving mice n=10; the experiment is designed for a two-sided alpha of 0.05, 80% power, with 10 mice per group. For Immunohistochemical studies a minimal a n=3 slides were analyzed and 5-8 fields per slide were counted or analyzed depending on the experiment. FACS studies, we used n=5 mice per group.
Data exclusions	N/A
Replication	All replication experiments were successful after standardizing tissue culture conditions, antibodies dilutions for western boots, FACS, IHC, and mutliplex IHC studies. We only observed a small variability in in vivo tumor growth, but with not a considerable difference.
Randomization	Mouse tumor tissues were randomly selected for immunotherapy studies, tumors for IHC studies were randomly selected for staining.
Blinding	All tumor IHC studies were blindly analyzed and evaluated by two independent scientist. RNAseq and Whole exome sequencing blinding was not relevant because the sequencing studies were performed by the Department of Bioinformatics.

Reporting for specific materials, systems and methods

We require information from authors about some types of materials, experimental systems and methods used in many studies. Here, indicate whether each material, system or method listed is relevant to your study. If you are not sure if a list item applies to your research, read the appropriate section before selecting a response.

Materials & experimental systems

n/a	Involved in the study
☐	☒ Antibodies
☐	☒ Eukaryotic cell lines
☒	☐ Palaeontology and archaeology
☐	☒ Animals and other organisms
☒	☐ Human research participants
☒	☐ Clinical data
☒	☐ Dual use research of concern

Methods

n/a	Involved in the study
☒	☐ ChIP-seq
☐	☒ Flow cytometry
☒	☐ MRI-based neuroimaging

Antibodies

Antibodies used

CD8a (Cell Signaling, 1:100, #98941)
CD4 (Cell Signaling, 1:100, #25229)
FoxP3 (eBioscience, 1:100, #14-5773-82)
CD68 (Abcam, 1:200, #ab125212)
CD11c (Cell Signaling, 1:100, #97585)
CD206 (Abcam, 1:2000, #ab64693)
PD-1 (Cell Signaling, 1:100, #84651)
CTLA-4 (Biorbyt Ltd, 1:500, #orb253158)
TIGIT (Millipore Sigma, 1:1500, #ZRB1454)
vimentin (Cell Signaling, 1:200, #5741)
Ki67 (Abcam, 1:1000, #ab15580)
cytokeratin 14 (ThermoFisher Scientific, 1:800, #PA5-167222).
Phycoerythrin (PE)-conjugated anti-EGFR antibody (GeneTex, #GTX20231)
PE-conjugated anti–IgG2a κ isotype control antibody (BioLegend, #400508)
anti-p53 (Cell Signaling, 1:1000, #32532)
β-actin (Santa Cruz Biotechnology, 1:5000, #sc81178)
CD45 (Tonbo, 1:100, #80-0454-U025)
CD3 (Biolegend, 1:100, # 100217)
CD4 (Biolegend, 1:100, # 100413)
CD8 (Biolegend, 1:100, # 100728)
CD19 (Tonbo, 1:100, # 20-0193-U025)
IFN-γ (Biolegend, 1:100, # 505805)
IL-4 (ThermoFisher, 1:100, #25-7042-41)
IL-17A (BD, 1:100, # 564171)
FoxP3 (ThermoFisher, 1:100, #12-5773-80)
PD-1 (Biolegend, 1:100, #748265)
CD11b (Biolegend, 1:100, #101205)
Ly6C (Biolegend, 1:100, #128015).
Ly6G (Biolegend, 1:100, # 127607)
CD206 (BioLegend, 1:100, #141717)
MHC II (BioLegend, 1:100, #107643)
Ghost Dye (Tonbo, 1:100, #13-0870-T100)

Validation

For IH or Multiplex, each antibody was validate perfoming control staining with secondary antibody only and using tissues expressing the corresponding targets. For antibodies binding to immune cells we used secondary lymph nodes to test primary antibodies.

Eukaryotic cell lines

Policy information about cell lines

Cell line source(s)

The mouse cell lines reported in this manuscript were generated in our lab

Authentication

The mouse cell lines were authenticated by IDEXX BioAnalytics.

Mycoplasma contamination

All cell lines tested negative for Mycoplasma contamination

Commonly misidentified lines
(See ICLAC register)

N/A

Animals and other organisms

Policy information about studies involving animals; ARRIVE guidelines recommended for reporting animal research

Laboratory animals

We used the following genetically engineered mouse models to generate the cell lines:
B6. K14Cre transgenic mice (Jackson Labs stock # 018964), Males and females 8-10 weeks old
B6. p53 knockout mice (Jackson Labs stock # 008462), Males and females 8-10 weeks old

B6. p53 R172H mutant mice (NCI mouse repository stock # 01XM2), Males and females 8-10 weeks old

For tumor growth studies we used:
C57BL/6 mice (Jacson Labs stock # 000664), Males and females 8-10 weeks old
Beige mice (Jackson Labs stock # 000629), Males and females 8-10 weeks old

Wild animals	The study did not include any wild animals
Field-collected samples	The study did not contain any samples collected from the field
Ethics oversight	All studies had been approved by the Institutional Animal Care and Use Committee at the University of Texas, MD Anderson Cancer Center

Note that full information on the approval of the study protocol must also be provided in the manuscript.

Flow Cytometry

Plots

Confirm that:

☒ The axis labels state the marker and fluorochrome used (e.g. CD4-FITC).

☒ The axis scales are clearly visible. Include numbers along axes only for bottom left plot of group (a 'group' is an analysis of identical markers).

☒ All plots are contour plots with outliers or pseudocolor plots.

☒ A numerical value for number of cells or percentage (with statistics) is provided.

Methodology

Sample preparation

Homogenize the spleen with frosted slides in petri dish and filter thru 70µm strainer back into RPMI. The bone marrow cells were harvested from femurs and tibias flushed with a 25G needle and filtered through a 70-µm cell strainer. Place 70µm cell strainer on top of 50mL tube and pour splenocytes or bone marrow cells onto strainer, allowing fluid to flow through. Add 10mL FACS buffer (going through strainer to rinse anything off) and centrifuge to pellet cells. Add 2mL/tube of RT 1X RBC Lysing Buffer and pulse vortex. Incubate 1min at 37°C, centrifuge (5 min at ~1400rpm at 4°C) and wash with FACS buffer. Resuspend cells in 10mL RPMI with 1% FBS, count cells.
Intracellular Cytokine Staining and FoxP3 for T Cells:
Add 2x106 cells/tube, including unstained and spleen controls; filter cells through 40µm strainer blue lid FACS tubes. Wash away RPMI and resuspend cells in large culture tube. In a 6 well plate, plate 3x106 cells/well per sample in 3mL complete RPMI Master Mix (include unstimulated control in complete RPM): 1µL/mL GolgiStop, 0.7µL/mL GolgiPlug, 0.05ug/mL PMA and 0.5 ug/mL ionomycin. Incubate 4 hours at 37°C. Collect cells from plate, rinsing 2 times with media until wells are visibly empty. Wash, then perform surface staining (omit Fc block for T cells). Fix with 100µL/sample of 1% formaldehyde in PBS for 20 min on ice. Wash twice to remove fixation buffer and re-suspend in 100µL FACS buffer. Store at 4°C.
Surface Staining:
Add 2x106 cells/tube, including unstained and spleen controls, filter cells through 40µm strainer blue lid FACS tubes. Wash and resuspend in anti-mouse CD16/32 Fc Shield (1µg/30µL/sample) for 20 min(omit for T cells). Without removing the Fc Shield, add 100µL/sample of antibody cocktail (diluted in FACS buffer) containing L/D and antibodies against surface markers. Incubate for 45mins at 4°C. Wash, Fix w/ 100µL/sample of 1% formaldehyde in PBS for 20 min on ice. Wash twice to remove fixation buffer and re-suspend in 150µL FACS buffer. Pellet cells, decant, and vortex (~ 100µL residual).
Intracellular/Intranuclear Staining:
Add 1mL FoxP3 Fixation/Permeabilization working solution/tube and vortex. Incubate for 45mins at 4°C. Wash twice with 2mL 1X Permeabilization Buffer. Add 100µL/sample of antibody cocktail (diluted in perm buffer) against intracellular makers. Incubate for 45mins at 4°C. Wash twice with 2mL 1X Permeabilization Buffer. Resuspend in 100µL FACS buffer.

Instrument	LSRFortessa X-20 flow cytometer (BD Biosciences)
Software	FlowJo software (Tree Star)
Cell population abundance	lymph cells compromised 60-70% of living cells. Myeloid cell compromised 85-95% of living cells. 97-99% of lymph cells and myeloid cell expressed of high-level of CD45.
Gating strategy	FSC-A/SSC-A was used to exclude debris, while SSC-H/SSC-W and FSC-H/FSC-W gates were used to remove duplets, FCS-A/ Fixable Viability stain (510) was used to deplete dead cells. Others described as Supplemental figure 7 and 8.

☒ Tick this box to confirm that a figure exemplifying the gating strategy is provided in the Supplementary Information.
